# Sleep is not just for the brain: transcriptional responses to sleep in peripheral tissues

**DOI:** 10.1186/1471-2164-14-362

**Published:** 2013-05-30

**Authors:** Ron C Anafi, Renata Pellegrino, Keith R Shockley, Micah Romer, Sergio Tufik, Allan I Pack

**Affiliations:** 1 Division of Sleep Medicine and Center for Sleep and Circadian Neurobiology, Perelman School of Medicine, University of Pennsylvania, Philadelphia, PA, USA; 2 Center for Applied Genomics, The Children’s Hospital of Philadelphia, Philadelphia, PA, USA; 3 Universidade Federal de São Paulo UNIFESP, São Paulo, Brazil; 4 Department of Health and Human Services, Biostatistics Branch, National Institute of Environmental Health Sciences, National Institutes of Health, Research Triangle Park, NC, USA

**Keywords:** Sleep, Circadian Rhythms, Sleep Deprivation, Unfolded Protein Response, Heart, Lung, Synchronization

## Abstract

**Background:**

Many have assumed that the primary function of sleep is for the brain. We evaluated the molecular consequences of sleep and sleep deprivation outside the brain, in heart and lung. Using microarrays we compared gene expression in tissue from sleeping and sleep deprived mice euthanized at the same diurnal times.

**Results:**

In each tissue, nearly two thousand genes demonstrated statistically significant differential expression as a function of sleep/wake behavioral state. To mitigate the influence of an artificial deprivation protocol, we identified a subset of these transcripts as specifically sleep-enhanced or sleep-repressed by requiring that their expression also change over the course of unperturbed sleep. 3% and 6% of the assayed transcripts showed “sleep specific” changes in the lung and heart respectively. Sleep specific transcripts in these tissues demonstrated highly significant overlap and shared temporal dynamics. Markers of cellular stress and the unfolded protein response were reduced during sleep in both tissues. These results mirror previous findings in brain. Sleep-enhanced pathways reflected the unique metabolic functions of each tissue. Transcripts related to carbohydrate and sulfur metabolic processes were enhanced by sleep in the lung, and collectively favor buffering from oxidative stress. DNA repair and protein metabolism annotations were significantly enriched among the sleep-enhanced transcripts in the heart. Our results also suggest that sleep may provide a *Zeitgeber*, or synchronizing cue, in the lung as a large cluster of transcripts demonstrated systematic changes in inter-animal variability as a function of both sleep duration and circadian time.

**Conclusion:**

Our data support the notion that the molecular consequences of sleep/wake behavioral state extend beyond the brain to include peripheral tissues. Sleep state induces a highly overlapping response in both heart and lung. We conclude that sleep enhances organ specific molecular functions and that it has a ubiquitous role in reducing cellular metabolic stress in both brain and peripheral tissues. Finally, our data suggest a novel role for sleep in synchronizing transcription in peripheral tissues.

## Background

Sleep is characterized by a reversible reduction in consciousness, an increase in arousal threshold, and a characteristic body posture [1]. Sleep is defined as a behavioral state and most theories as to the function of sleep have naturally focused on the brain. The synaptic homeostasis theory [2], the brain energetic restoration theory [3,4], the memory consolidation theory [5]; and the macromolecular biosynthesis theory [6] have all placed a central emphasis on the importance of sleep for the brain. Indeed Hobson famously declared sleep to be “of the brain, by the brain, and for the brain” [7].

Yet, the effects of sleep and sleep deprivation on the body at large are hard to ignore [8]. In humans there are well characterized changes in metabolism [9] and hormone secretion [10,11] associated with sleep and sleep deprivation. Sleep state is known to impact the pathophysiology of several diseases including asthma, and vaccine based studies suggest that sleep can alter immune function [12,13].

Over the last decade the functions of sleep have been explored through microarray studies [14]. These studies have evaluated the nature of the genes in specific brain regions whose expression varies between sleep and wake and with sleep deprivation. Such studies have been conducted in rats [15], mice [16], Drosophila [17,18], and sparrows [19]. The results of these studies have, in many ways, spurred the development of the theories discussed above [14] and have been combined in a recent meta-analysis [20]. Statistical and experimental differences among these studies, particularly in sample size, led to variation in the absolute number of differentially expressed transcripts identified. However, taken together, these studies point to several key pathways being regulated in the brain as a function of sleep/wake behavioral state. Cholesterol synthesis [6] for example is relatively up-regulated during sleep while genes involved in synaptic plasticity such as *Bdnf*, *Arc*, *Homer 1a *[16] are up-regulated during wakefulness. Moreover, in all species studied to date, deprivation of sleep leads to up-regulation of molecular chaperones [15-20] suggesting that sleep deprivation leads to endoplasmic reticulum (ER) stress in brain with activation of the unfolded protein response [21,22].

By design, these studies have all been focused on changes in gene expression in the brain. The seminal study of Maret et al. included microarray profiling of both brain and of liver and is, to date, the only study to evaluate genome wide changes in expression in a peripheral organ as a function of sleep state [16]. In that study, sleep deprivation induced statistically significant expression changes in nearly three times as many genes in liver as compared to brain. Moreover, mouse strains that showed different susceptibilities to sleep deprivation and differential transcriptional responses to sleep loss in whole brain also showed different transcriptional responses in the liver. However, Maret et al. specifically used changes observed in liver to help evaluate the changes observed in the brain, assuming that gene expression changes in liver are only tangential to the function of sleep. The transcriptional changes induced by sleep and sleep deprivation in other tissues remain generally unexplored.

Here we report the molecular consequences of sleep and sleep deprivation in lung and heart. We probed the transcriptional differences between sleeping and sleep-deprived states and further challenged the concept that the molecular consequences of sleep state are primarily limited to the brain. Matched animals from both groups were sacrificed at the same circadian times to control for time of day effects. Groups were euthanized after 4 different durations of sleep and sleep deprivation. We identified those transcripts that had both significantly different expression as a function of sleep/wake behavioral state (sleep compared to sleep deprivation) and showed a definite positive or negative change with time in the spontaneously sleeping groups. Our data indicate that sleep state has a marked transcriptional effect in peripheral tissues. Sleep reduces markers of ER stress in both heart and lung and sleep enhances metabolic process in an organ specific fashion. Our data also suggest that sleep plays a role synchronizing transcription of certain pathways in the lung.

## Results and discussion

**Figure 1:**
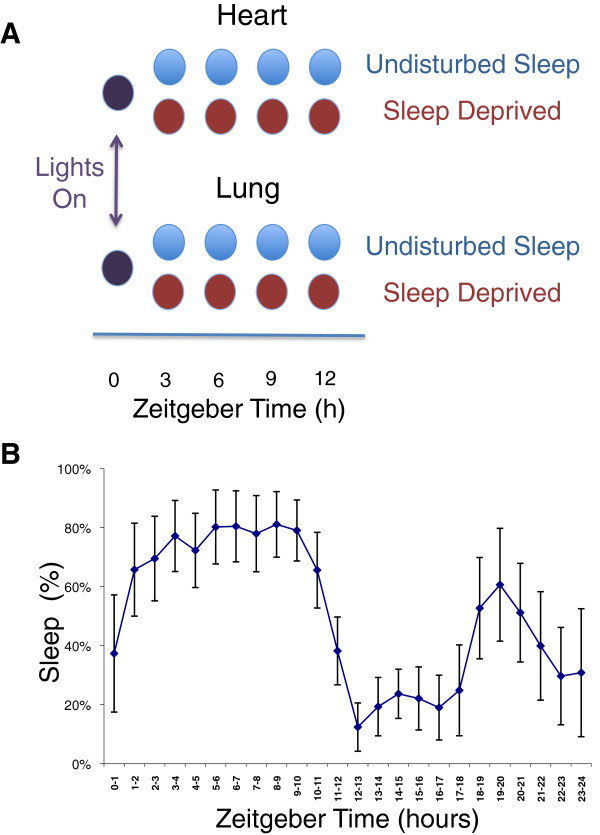
**Experimental design.** The experiment compared gene expression in sleep and sleep-deprived animals in two peripheral organs (heart and lung). **(A)**: Groups of mice were either allowed uninterrupted sleep or were sleep-deprived via gentle handling. Baseline animals were sacrificed at zeitgeiber time 0 (ZT0 - lights on) and diurnally matched animals from both groups were sacrificed after 3, 6, 9, and 12 hours. Animals were only fed during the lights-off (dark) period for 14 days prior to study to prevent differential food intake. **(B)**: Activity was monitored by a non-invasive locomotor monitoring system. Sleep was considered to be present when there was 40 seconds or more of continuous inactivity. Sleep was assessed in undisturbed control mice across the entire 24 hour cycle. The percentage of each hour that control mice slept is plotted as a function of time. Data shown are mean ± standard deviation. In the three-hour-blocks preceding sample collection at ZT3, ZT6, ZT9, and ZT12, animals in the undisturbed sleep group slept for 58%, 77%, 79%, and 61%, respectively, of the available sleep opportunity.

Given the established consequences of sleep deprivation on the immune [12,13,23],cardiovascular [12,24], metabolic [9,11,25,26] and musculoskeletal function [27], It is reasonable to suspect that sleep state impacts a broad array of peripheral tissues. We evaluated the molecular consequences of sleep and sleep deprivation on two peripheral tissues -- lung and heart. While this choice of tissues is somewhat arbitrary, these tissues have undeniable physiologic importance. Moreover the rhythmic beating of the heart, like breathing, continues unabated throughout the entire sleep wake cycle. Thus the direct influence of sleep on these tissues presents an important test of ubiquitous influence of sleep/wake behavioral state on molecular physiology. We used microarrays to compare the gene expression in sleeping and sleep-deprived mice euthanized at the same diurnal time (see Figure 1).

The animals used in this study were also used in a previously published study investigating the transcriptional impact of sleep and sleep deprivation on gene expression in different brain regions [6]. Heart and lung tissues were obtained along with brain at the time of animal sacrifice. The details of the experimental method and behavioral analysis have been reported previously [6]. In brief, male mice (n = 80, C57BL/6 J) were housed in a 12 h: 12 h light/dark cycle (7:00 am/7:00 pm). All mice were acclimated to the experimental environment for 14 days prior to the study. During this period, mice were fed only at night to avoid differential food intake between sleeping and sleep-deprived mice during the day of study. Ten undisturbed animals were euthanized at lights on (7:00 am) to establish baseline transcript expression. The remaining animals were split into two main groups: one group was allowed uninterrupted sleep opportunity while the other group underwent continued sleep deprivation. The fraction of the sleep opportunity during which the control animals slept (as assessed by activity measured from electronic beam breaks) is shown in Figure 1B. Deprivation through gentle handling was initiated at lights-on. Eight or nine animals were subsequently euthanized from each of the two groups (sleep deprived and uninterrupted sleep opportunity) after 3, 6, 9, and 12 hours from lights on. Total RNA was isolated from heart and lung samples. Transcript levels were assayed with the GeneChip® Mouse Gene 1.0 ST array (Affymetrix, Santa Clara, CA). Experimental costs limited the number of tissues samples from brain that were profiled in the previous study [6]. As a result, the data for a larger number of animals and microarray results are reported here. One of the primary limitations of our study, like other microarray studies of sleep, is that we do not distinguish the transcriptional effects of sleep from those imparted by isolated physical rest. For simplicity we refer to sleep induced changes. However, in the work that follows it is important to note that the relative contributions of sleep and physical rest remain uncertain.

### Sleep and enforced sleep deprivation have a marked and coordinated impact on transcription in lung and heart

We identified genes in lung and heart that were differentially expressed as a function of behavioral state at an FDR (false-discovery rate) cutoff of 1%. Of the 35,556 probe sets and 20,406 unique named genes annotated on the array, 2470 genes (~12%) were differentially expressed between sleeping and sleep-deprived mice in the heart, while 1922 genes were differentially expressed in lung. Thus, significant fractions of genes in peripheral tissues are affected by sleep and/or sleep deprivation. Moreover, the degree of overlap in the lists of differentially expressed genes for the heart and lung is more than expected by chance (p < 2.2 10E-16 by Fisher’s exact test), suggesting a common function and/or regulation.

Many of the genes identified as being differentially expressed in these tissues were also found to be differentially expressed in brain in previous studies [15-20], again suggesting that behavioral state imparts coordinated transcriptional changes throughout the whole organism. A recent meta-analysis of transcriptional changes in brain by Wang *et al. *[20] enumerated 93 unique named genes that have been identified in multiple studies as being differentially expressed as a function of sleep state in different brain regions and in different species. This number of genes probably underestimates the expression changes induced by sleep/wake behavioral state in the brain given the limited statistical power of some of the component studies. Nonetheless, of these 93 genes, we found 51 (55%) were also differentially expressed in heart and/or lung at an FDR of 1%. Again, this degree of overlap is more than would be expected from random chance (p < 2.2 10E-16 by Fisher’s exact test), especially considering that many genes expressed in brain are not expressed in these peripheral tissues.

### Defining “strict sleep enhanced/repressed genes” highlights transcriptional changes that result from sleep/wake state as opposed to deprivation protocols

Sleep deprivation studies have been criticized with the concern that observed changes may reflect the consequence of a particular artificial deprivation protocol rather than natural sleep/wake physiology [16]. Indeed it can be similarly argued that extended sleep deprivation may induce physiological responses that are distinct from, as opposed to an augmentation of, the responses to normal wakefulness. Moreover, a direct comparison between sleep and sleep-deprived expression alone would not permit us to assess whether sleep or sleep deprivation induced the observed expression changes. To better address these concerns, we supplemented the above analysis to include baseline data from the animals sacrificed at lights-on. This additional baseline time point reflects gene expression immediately prior to the initiation of the major sleep period. For each transcript that had significant differential expression between sleep and sleep deprivation, linear regression was used to fit the time course of transcript expression in unperturbed animals starting with baseline expression and continuing with expression in spontaneously sleeping mice sacrificed after 3, 6, 9 and 12 hours. We then defined “strict sleep enhanced” or “strict sleep repressed” genes as those differentially expressed genes that also had a significant non-zero slope of gene expression with time (FDR <1%) among the non-perturbed sleeping animals. The use of regression was not intended as a test of a true *linear* pattern, but rather as a tool to assess the general temporal trend in expression over time with undisturbed sleep. The direction of the temporal changes induced by spontaneous sleep was required to be commensurate with the direction of change observed when comparing the naturally sleeping and sleep-deprived mice. Thus, to identify a gene as a “strict sleep enhanced gene” we required both that its transcription be significantly greater in sleeping animals when compared to circadian matched sleep-deprived animals and that transcript levels increase over the time course of the natural sleep period. The converse criteria were required of genes said to be strict sleep repressed genes. By restricting our attention to genes that were observed to also have temporal expression changes among undisturbed, spontaneously sleeping animals, we safeguarded against the possibilities that the changes were solely the result of stress due to gentle handling or an artificial facet of the deprivation protocol.

Using these strict criteria, a total of 573 genes in the lung and 1182 genes in the heart showed sleep specific changes. These genes are listed in the Additional file 1. Thus 3% and 6% of the assayed transcripts showed “sleep specific” changes in the lung and heart respectively and there was a significant overlap between the two tissues (156 probe sets, 139 unique genes, p < 2.2 10E-16 by Fisher’s exact test).

**Figure 2:**
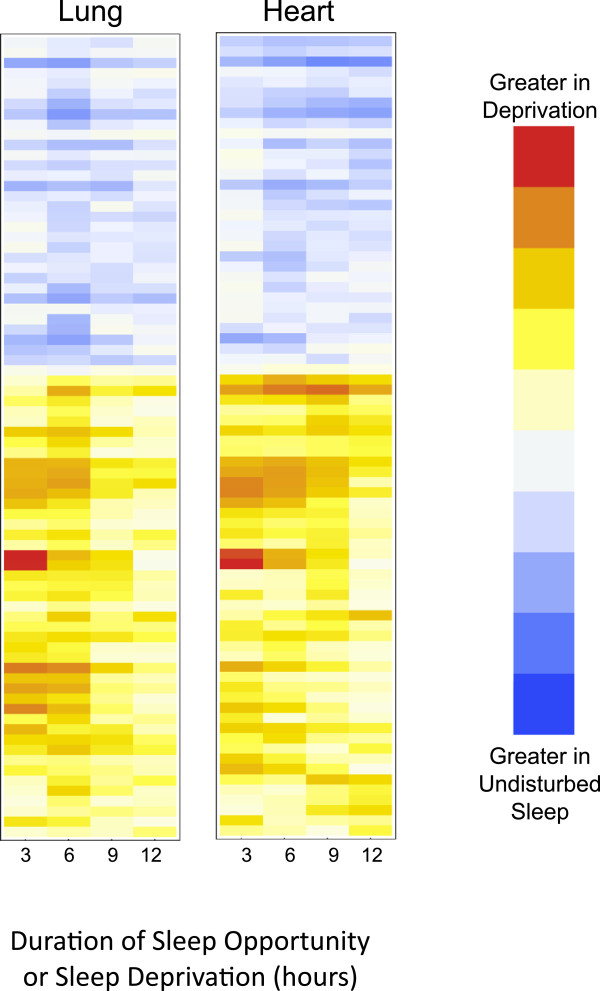
**Dynamics of differential expression in heart and lung.** The difference in transcript expression between sleeping and diurnally matched, sleep-deprived animals is a function of deprivation time. This heat map shows the difference in mean transcript levels between sleeping and sleep-deprived mice (in log space), as sleep deprivation continues. Darker shades represent increasing magnitudes of differential expression. Red and blue signify increased expression in sleep deprived and spontaneously sleeping animals respectively. All 156 probes that had sleep specific changes in both tissues are displayed. Dynamic changes in the lung are plotted alongside dynamic changes in the heart for the same probe. The direction of change and the temporal evolution of those changes in the heart and lung are similar.

Further suggestion that sleep state imparts a coordinated effect on peripheral transcription can be found in noting the similarity in the temporal profiles of the sleep specific transcripts in both tissues. As shown in Figure 2, we compared the difference (in log space) between gene expression in sleeping and sleep-deprived animals as a function of time. An agglomerative clustering algorithm was used to order transcripts based on the temporal pattern and results are plotted as a heat map. The temporal progression for each gene in the lung is plotted alongside the results for the same gene in the heart. Not only was the direction of differential expression highly consistent across the tissues, there is also a marked similarity in temporal progression.

To generate hypotheses as to the molecular consequences of sleep in peripheral tissues we uploaded the four lists (strict sleep enhanced and repressed transcripts in the lung and heart) to the NIH Database for Annotation, Visualization and Integrated Discovery (DAVID) data analysis suite (http://david.abcc.ncifcrf.gov) [28,29]. We evaluated these lists for overrepresentation in one of the following categories: gene ontology (GO) biological process, Protein Information Resource (PIR) Key words, and Kyoto Encyclopedia of Genes and Genomes (KEGG) pathways. The DAVID functional clustering tool was used to group overlapping biological terms or pathways into clusters that likely reflect common physiology. The results are shown in Tables 1, 2, 3, and 4. Tables 1 and 2 show the functional annotations enriched among the sleep repressed genes in the heart and lung respectively. Only annotations enriched at an FDR <5% are retained. Tables 3 and 4 show the functional annotations enriched among sleep enhanced genes in these same tissues. In both tissues, sleep specific transcriptional changes demonstrated significant enrichment for various physiologic processes.

**Table 1:** Significantly overrepresented functional annotations describing sleep-repressed transcripts in the heart

	**Cluster 1**			
**Category**	**Term**	**P-Value**	**Fold Enrichment**	**FDR(%)**
SP_PIR_KEYWORDS	Chaperone	7.39E-09	4.992	1.0E-05
SP_PIR_KEYWORDS	Stress response	3.39E-08	9.446	4.6E-05
GOTERM_BP_FAT	GO:0006457 Protein folding	5.87E-05	3.908	1.0E-01
	**Cluster 2**			
**Category**	**Term**	**P-Value**	**Fold Enrichment**	**FDR(%)**
GOTERM_BP_FAT	GO:0043066 Negative regulation of apoptosis	1.13E-06	3.400	0.00195
GOTERM_BP_FAT	GO:0043069 Negative regulation of programmed cell death	1.61E-06	3.330	0.00277
GOTERM_BP_FAT	GO:0060548 Negative regulation of cell death	1.73E-06	3.316	0.00298
GOTERM_BP_FAT	GO:0042981 Regulation of apoptosis	4.09E-05	2.173	0.07051
GOTERM_BP_FAT	GO:0043067 Regulation of programmed cell death	5.27E-05	2.145	0.09073
GOTERM_BP_FAT	GO:0010941 Regulation of cell death	5.85E-05	2.133	0.10074
GOTERM_BP_FAT	GO:0006916 Anti-apoptosis	2.01E-04	4.361	0.34531
	**Cluster 3**			
**Category**	**Term**	**P-Value**	**Fold Enrichment**	**FDR(%)**
GOTERM_BP_FAT	GO:0022604 Regulation of cell morphogenesis	1.79E-05	4.725	0.03093
	**Cluster 4**			
**Category**	**Term**	**P-Value**	**Fold Enrichment**	**FDR(%)**
SP_PIR_KEYWORDS	Nucleosome core	7.18E-04	6.358	0.97486

Sleep repressed transcripts demonstrate both significantly reduced expression in sleep as compared to sleep deprivation (FDR < 1%) and a decreasing expression as spontaneous sleep progresses (FDR <1%). Overrepresented functional annotations describing this group of transcripts that met a FDR cutoff of 5% are shown. Clusters describe overlapping gene annotations as determined by the DAVID functional clustering feature.

**SP_PIR:** Swiss-Prot Protein Information Resource; **GOTERM_BP_FAT**: Gene ontology term Biological process; **KEEG:** Kyoto Encyclopedia of Genes and Genomes.

**Table 2:** Significantly overrepresented functional annotations describing sleep-repressed transcripts in the lung

	**Cluster 1**			
**Category**	**Term**	**P-Value**	**Fold Enrichment**	**FDR(%)**
SP_PIR_KEYWORDS	Chaperone	4.07E-15	10.133	5.40E-12
SP_PIR_KEYWORDS	Stress response	4.51E-14	21.351	5.94E-11
SP_PIR_KEYWORDS	Molecular chaperone	4.92E-12	44.118	6.46E-09
GOTERM_BP_FAT	GO:0006457 Protein folding	7.28E-11	8.835	1.18E-07
	**Cluster 2**			
**Category**	**Term**	**P-Value**	**Fold Enrichment**	**FDR(%)**
SP_PIR_KEYWORDS	Molecular chaperone	4.92E-12	44.118	6.46E-09
SP_PIR_KEYWORDS	Stress-induced protein	0.001235	51.471	1.611722
SP_PIR_KEYWORDS	Heat shock	0.001235	51.47	1.611722
	**Cluster 3**			
**Category**	**Term**	**P-Value**	**Fold Enrichment**	**FDR(%)**
SP_PIR_KEYWORDS	Isomerase	0.00167	4.65	2.18
	**Cluster 4**			
**Category**	**Term**	**P-Value**	**Fold Enrichment**	**FDR(%)**
KEGG_PATHWAY	NOD-like receptor signaling pathway	2.78E-04	7.537	0.30520
	**Cluster 5**			
**Category**	**Term**	**P-Value**	**Fold Enrichment**	**FDR(%)**
KEGG_PATHWAY	Antigen processing and presentation	3.99E-05	6.840	0.04383

Sleep repressed transcripts demonstrate both significantly reduced expression in sleep as compared to sleep deprivation (FDR < 1%) and a decreasing expression as spontaneous sleep progresses (FDR <1%). Overrepresented functional annotations describing this group of transcripts that met a FDR cutoff of 5% are shown. Clusters describe overlapping gene annotations as determined by the DAVID functional clustering feature.

**SP_PIR:** Swiss-Prot Protein Information Resource; **GOTERM_BP_FAT**: Gene ontology term Biological process; **KEEG:** Kyoto Encyclopedia of Genes and Genomes.

**Table 3:** Significantly overrepresented functional annotations describing sleep-enhanced transcripts in the heart

	**Cluster 1**			
**Category**	**Term**	**P-Value**	**Fold Enrichment**	**FDR(%)**
GOTERM_BP_FAT	GO:0044265 Cellular macromolecule catabolic process	1.1E-06	2.259	0.002
GOTERM_BP_FAT	GO:0019941 Modification-dependent protein catabolic process	3.8E-06	2.328	0.006
GOTERM_BP_FAT	GO:0051603 Proteolysis involved in cellular protein catabolic process	4.5E-06	2.278	0.008
GOTERM_BP_FAT	GO:0044257 Cellular protein catabolic process	5.2E-06	2.266	0.009
GOTERM_BP_FAT	GO:0009057 Macromolecule catabolic process	7.0E-06	2.102	0.012
GOTERM_BP_FAT	GO:0030163 Protein catabolic process	1.1E-05	2.188	0.019
SP_PIR_KEYWORDS	Ublquitin conjugation pathway	1.9E-05	2.242	0.025
	**Cluster 2**			
**Category**	**Term**	**P-Value**	**Fold Enrichment**	**FDR(%)**
SP_PIR_KEYWORDS	Zinc-finger	8.5E-04	1.543	1.147
SP_PIR_KEYWORDS	Zinc	1.5E-03	1.393	2.007
SP_PIR_KEYWORDS	Metal-binding	2.9E-03	1.293	3.845
	**Cluster 3**			
**Category**	**Term**	**P-Value**	**Fold Enrichment**	**FDR(%)**
GOTERM_BP_FAT	GO:0006259 DNA metabolic process	3.0E-05	2.360	0.050
GOTERM_BP_FAT	GO:0006281 DNA repair	2.2E-04	2.732	0.363
GOTERM_BP_FAT	GO:0006974 Response to DNA damage stimulus	2.8E-04	2.444	0.463
SP_PIR_KEYWORDS	DNA damage	5.5E-04	2.720	0.743
SP_PIR_KEYWORDS	DNA repair	1.4E-03	2.693	1.914
	**Cluster 4**			
**Category**	**Term**	**P-Value**	**Fold Enrichment**	**FDR(%)**
SP_PIR_KEYWORDS	RNA-binding	1.4E-07	2.568	2E-04
KEGG_PATHWAY	Spliceosome	7.3E-05	3.757	9E-02

Sleep enhanced transcripts demonstrate both significantly increased expression in sleep as compared to sleep deprivation (FDR < 1%) and increasing expression as spontaneous sleep progresses (FDR <1%). Overrepresented functional annotations describing this group of transcripts that met a FDR cutoff of 5% are shown. Clusters describe overlapping gene annotations as determined by the DAVID functional clustering feature.

**Table 4:** Significantly overrepresented functional annotations describing sleep-enhanced transcripts in the lung

	**Cluster 1**			
**Category**	**Term**	**P-Value**	**Fold Enrichment**	**FDR**
SP_PIR_KEYWORDS	Carbohydrate metabolism	6.82E-05	7.856	0.089071
	**Cluster 2**			
**Category**	**Term**	**P-Value**	**Fold Enrichment**	**FDR**
SP_PIR_KEYWORDS	Glycoprotein	5.49E-04	1.445	0.714887
	**Cluster 3**			
**Category**	**Term**	**P-Value**	**Fold Enrichment**	**FDR**
GOTERM_BP_FAT	GO:0006790 Sulfur metabolic process	0.0015	5.604	2.36

Sleep enhanced transcripts demonstrate both significantly increased expression in sleep as compared to sleep deprivation (FDR < 1%) and increasing expression as spontaneous sleep progresses (FDR <1%). Overrepresented functional annotations describing this group of transcripts that met a FDR cutoff of 5% are shown. Clusters describe overlapping gene annotations as determined by the DAVID functional clustering feature.

### Sleep reduces markers of ER stress and the unfolded protein response in both tissues

**Figure 3:**
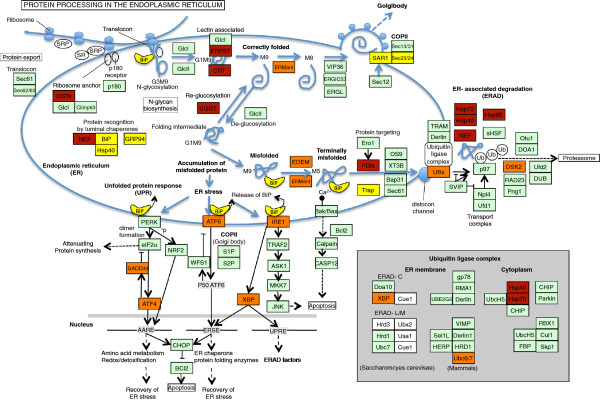
**Sleep specific effects on protein processing and the unfolded protein response.** KEGG Pathway diagram depicting protein processing in the endoplasmic reticulum. Sleep repressed genes common to both tissues are colored maroon. Sleep repressed genes in the heart (or lung) alone are colored orange (or yellow).

**Figure 4:**
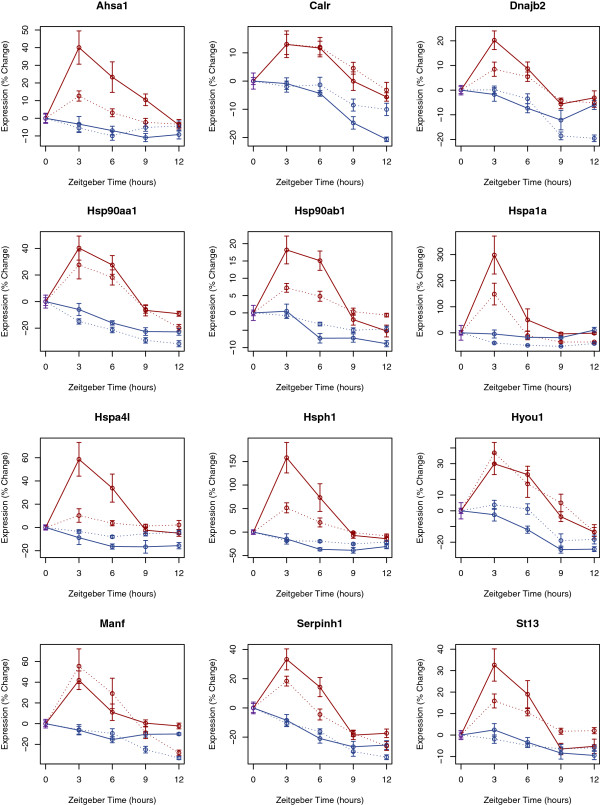
**Transcript profiles of sleep repressed genes involved in the unfolded protein response.** Temporal profiles of expression for select sleep responsive genes with annotations relating to protein processing and endoplasmic reticulum (ER) stress. Changes in gene expression from the baseline condition (lights on) are plotted as a percent of baseline expression levels. Maroon curves show data from experimentally sleep deprived animals and blue curves show data from spontaneously sleeping animals. Data from heart tissue is connected with dashed lines. Data from lung tissue is connected by uninterrupted lines. All genes shown here met statistical criteria for sleep specific repression in both tissues. Data shown represents mean ± standard error for 8 or 9 biological replicates.

There were 281 sleep-repressed genes in the lung and 639 sleep repressed genes in the heart. The DAVID analysis of both of these lists (Tables 1 and 2) showed a prominent overlap in the top categories of enrichment: Chaperone proteins, endoplasmic reticulum (ER)-related transcripts, and heat shock proteins were all highly overrepresented. Relative to wake and enforced deprivation, sleep reduces the transcription of key chaperone proteins. Notably these categories actually represent highly overlapping biological features involved in the protein folding and the Unfolded Protein Response (UPR). Figure 3 shows the annotated KEGG pathway describing protein processing. Sleep repressed transcripts in the heart are shown in orange, sleep repressed transcripts in the lung are shown in yellow, and sleep repressed transcripts common to both are shown in red. As already noted, the relative up-regulation of the UPR with wakefulness has been a prominent finding among transcriptional studies in the brain. Thus, the transcriptional pathways affected by sleep are shared, in part, between heart, lung, and brain. Indeed sleep/wake behavioral state influences key molecules throughout the pathway. Expression profiles for several of the genes repressed by sleep in both the heart and lung that have annotations related to ER stress are shown in Figure 4.

The UPR was first connected to the molecular response to sleep deprivation in the fly brain [30] and has since been found to be up-regulated by sleep deprivation in different brain regions across multiple species (for review, see [14]). In the brain, the differential expression of molecular chaperones and the UPR between sleep and sleep deprivation is maintained in mice after adrenalectomy [31] and is thus not the result of HPA axis stress. The UPR was also up-regulated with extended wakefulness in the liver [16], and PCR confirmed that sleep deprivation enhanced the transcription of *BiP* (Immunoglobin binding protein), a master regulator of the UPR, in skeletal muscle [32].

The UPR is a highly conserved response to cellular metabolic stress and the accumulation of misfolded proteins (for reviews, see [33,34]). This three-armed response to the accumulation of misfolded proteins involves: (1) reducing further protein translation to avoid further overtaxing the endogenous chaperones, (2) up-regulating the transcription of chaperones to assist in proper protein folding, and (3) inducing apoptosis if cellular homeostasis cannot be restored [34]. In mouse cerebral cortex, all three arms of the unfolded protein response occur and are up-regulated after 6 hours of sleep deprivation [22]. Diverse metabolic challenges can all ultimately induce the UPR [34]. It is unknown if the metabolic alterations induced by sleep loss are the same in heart, lung, and brain. The metabolic insults may reflect the unique demands of each tissue. Regardless of the inciting mechanism, the common downstream consequences of the UPR and the resulting reduction in protein translation are expected to impact these peripheral tissues as well as brain. It is also worth noting that while several genes in the UPR pathway met the strict criteria for sleep repressed genes in both tissues, a larger set met the criteria in only one. For example, *BiP* (official symbol *Hspa4*, Heat shock protein 4) met criteria for a sleep repressed transcript in the lung. In the heart, *BiP* expression showed a reduction in sleep relative to enforced sleep deprivation but this reduction did not meet the pre-defined level of significance.

### Sleep enhances molecular processes in an organ specific manner

Using DAVID, we identified KEGG pathways and GO biologic categories overrepresented among the strict sleep enhanced genes in the lung: carbohydrate metabolism, glycoproteins and sulfur metabolism.

These classes are of broad physiologic importance and the global metabolic consequences of the sleep-induced changes are thus difficult to simply characterize. However, more focused attention to the particular genes involved suggests that sleep-dependant processes are important in the regulation of oxidative stress in the lung. For example, Pyruvate dehydrogenase kinase isozyme 1 (*Pdk1*) is among the many sleep-enhanced genes involved in carbohydrate metabolism. Pdk1 phosphorylates, and thereby inactivates, the mitochondrial pyruvate dehydrogenase complex that catalyzes the rate-limiting step in the initiation of the Citric Acid, or Krebs, cycle [35]. Thus, the translation of *Pdk1* serves to slow a major checkpoint of oxidative metabolism.

Sulfur metabolism is a similarly ubiquitous biological process, yet the sleep-enhanced transcripts in these pathways also appear to protect the lung in the face of oxidative stress and free radicals. Glutathione S-transferase 1 and 2 (*Gstt1* and *Gstt2*) were up-regulated with sleep and are part of a multigenic family responsible for the conjugation of oxidized, toxic, molecules with reduced glutathione [36]. Thioredoxin reductase 3 (*Txnrd3*), another sleep enhanced transcript, is an enzyme with glutathione and thioredoxin reducing activity – and thus enhances the ability to buffer oxidative free radicals [37]. Taken together these changes will serve to slow the generation of oxidative stressors and free radicals, increase the generation of reducing agents, and enhance the conjugation of these scavengers with extant free-radicals.

There is a long history of studies connecting sleep with the response to oxidative stress. Oxidized glutathione itself has been shown to have somnogenic properties [38] and sleep deprivation has previously been reported to decrease glutathione protein levels in both brain [39] and liver [40]. Mackiewicz et al. showed increased expression of antioxidant enzymes during sleep in the hypothalamus [6]. While these results are in contrast with Ramanathan’s report of an increase in brain anti-oxidant responses in the setting of short term sleep deprivation [41], the bulk of the data suggest that sleep is a time for the brain to buffer the oxidative load imposed by wakefulness. The lung, like the brain, is subject to considerable oxidative stress [6,42], and sleep appears to also provide the lung with a temporal compartment favoring the electrochemical reduction of free radicals.

### Sleep enhances targeted protein breakdown and DNA repair in the heart

Vagal tone and the baroreflex contribute to cardiac control and change during both REM and NREM sleep [43,44]. As a result, heart rate and blood pressure decline during NREM sleep and the heart has a reduced workload [44]. Surprisingly, the most significantly enriched annotations among the strict sleep enhanced genes in the heart related to the catabolism or breakdown of macromolecules. Sleep enhanced transcripts in the heart were enriched for the ubiquitin–proteasome system (UPS) pathway (Additional file 2: Figure S1). The UPS tags intracellular proteins by conjugation with ubiquitin and marks them for degradation [45]. We observed sleep enhanced expression of several Fbox family molecules including *Fbxo11*, *Fbxo3*, *Fbxo47* and *Fbxo1*. These Fbox proteins are components of Skp, Cullin, F-box (SCF) containing complex that catalyzes the ubiquitination of target proteins [46]. E3 ligases that covalently attach ubiquitin to specific target proteins also showed sleep enhanced transcription. Enhanced transcription of the machinery for protein degradation is at odds with our initial hypothesis that sleep is an anabolic period. However, targeted protein breakdown is distinct from generalized protein degradation. We speculate that, in the heart, sleep may provide a temporal window for the targeted breakdown of errant peptides. Targeted breakdown reflects a checkpoint where damaged proteins and now un-needed regulatory factors may be removed prior to new synthesis [45]. In addition, the UPS maintains the quality of nascent proteins emerging from the ER [47]. This hypothesis requires further experimental verification, but is consistent with the sleep induced down regulation of the UPR. The removal of damaged peptides during sleep would prevent their accumulation and cellular stress.

The second functional cluster overrepresented among sleep-enhanced genes in the heart involves DNA repair and regulation. The adult mammalian cardiomyocyte cell is a “textbook example” of a terminally differentiated, non-dividing cell [48]. While growing evidence suggests that there is cardiomyocyte turnover in the adult heart, the slow pace of division (<2% per year), suggests that DNA synthesis is not a major metabolic demand on the cardiomyocyte [49]. In that context, the importance of DNA metabolism appears questionable. However, DNA damage is a well characterized consequence of both mechanical and oxidative stress in cardiomyocytes [50,51]. Increasing DNA damage likely impedes proper transcription and evidence of DNA damage is associated with the progression to heart failure regardless of the underlying etiology [52]. Thus repair of DNA in the relatively fixed number of cardiomyocytes places a unique burden on the heart [51].

*Ogg1*, one of the sleep enhanced transcripts, has been specifically shown to function in the excision repair of DNA in cardiomyocytes from human patients with end stage cardiomyopathy as well as from mouse models of cardiac stress [52]. Decreased levels of *Brca2*, another sleep induced gene involved in DNA repair, have been shown to worsen doxorubicin induced heart failure [53]. DNA repair is an essential requirement for the continued health of the myocardium. While a circadian rhythm in DNA repair has already been described in other cells [54,55], in the heart this essential function is augmented by sleep.

### Sleep state effects circadian clock expression in the lung

**Figure 5:**
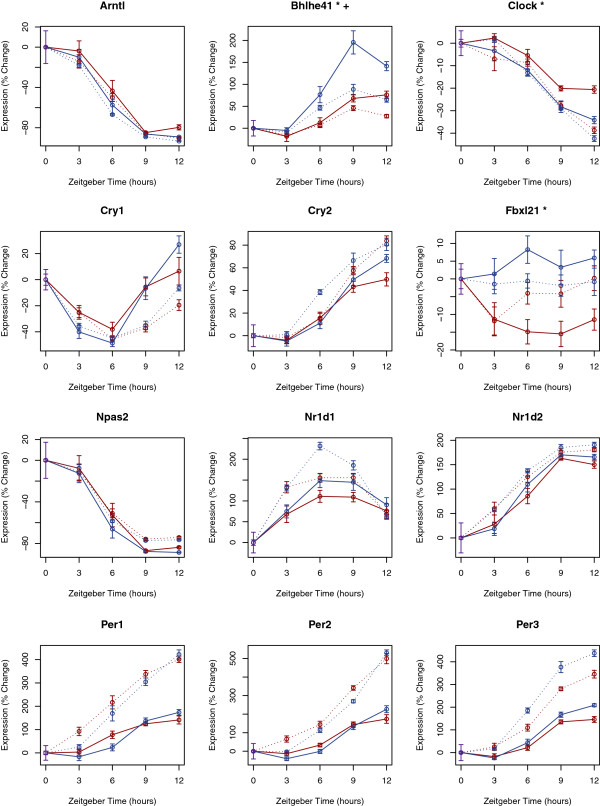
**Effect of sleep and sleep deprivation on the expression of core clock components.** The expression of exemplar circadian genes is plotted as a function of time in both the heart and lung. Changes in transcript expression from baseline conditions (lights on) are plotted as a percent of baseline expression levels. Maroon curves show data from experimentally sleep-deprived animals and blue curves show data from spontaneously sleeping animals. Data from heart tissue is connected with dashed lines. Data from lung tissue is connected by uninterrupted lines. Transcripts annotated with a (*) or (+) showed differential expression in the lung or heart respectively (p < 0.001). Data shown represents mean ± standard error for 8 or 9 biological replicates.

Discrete and independent circadian and homeostatic processes are believed to regulate sleep. Yet recent evidence suggests that at the molecular level these processes are intertwined [56]. At its core, the molecular circadian pacemaker is driven by the CLOCK/BMAL transcriptional activator complex. In the cerebral cortex the expression of several canonical circadian genes increases with sleep deprivation [57,58]. Moreover, a variant discovered in the *Dec2* gene (official symbol *Bhlhe41*), a modulator of CLOCK/BMAL mediated transcription [59], has been shown to influence sleep need [60]. Separately, recent work from the Takahshi and Schibler labs has demonstrated that small changes in temperature, as are expected from the normal physiology of sleep, are powerful time cues for peripheral oscillators [61-63]. Cold induced RNA binding protein (*Cirbp*) has been implicated in this effect [64] and *Cirbp* demonstrated sleep enhanced transcription in both heart and lung. Maret et al. similarly noted sleep induced changes in liver *Cirbp expression*[16], Thus we questioned if sleep and sleep deprivation specifically influence the expression of core clock genes in heart and lung. Most core circadian genes were unaffected by sleep state (Figure 5). However, *Dec2 *[59] (official symbol *Bhlhe41*), *Clock* and *Fbxl21* (which participates in the regulated degradation of some clock components) [65-67], all showed significant differential expression between sleep and sleep deprivation (p < 0.001) in the lung. *Dec2* expression was also significantly affected by behavioral state in the heart, but with a smaller fold-change. Given the increase in Clock and decrease in *Dec2* expression observed with sleep deprivation, we expected an increase in the canonical, E-box driven, clock output genes *Dbp*, *Tef*, and *Hlf *[68]. Unexpectedly, but in accord with previous observations in the cerebral cortex [69], *Dbp* transcript levels fell during sleep deprivation. In the cortex this decrease has been ascribed to reduced binding of BMAL1 and NPAS2 (the functional paralogue of CLOCK) to the *Dbp* promoter with sleep deprivation [69].

### Sleep synchronizes gene expression in the lung

**Figure 6:**
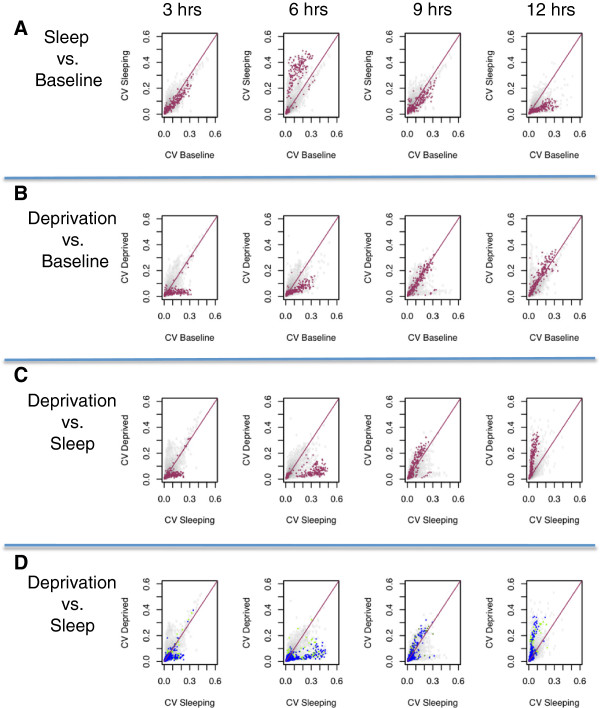
**Effect of sleep and sleep deprivation on the inter-animal variability of transcript expression in the lung.** 210 probes showed statistically significant changes in expression variability as a function of sleep duration. **(A)**: The coefficient of variation (CV) of probe expression among spontaneously sleeping mice after 3, 6, 9, and 12 hours is plotted against the CV of the same probe at baseline (lights-on). The red line of identity (x = y) demarcates no change in inter-animal variability. Probes with statistically significant temporal changes in variance among sleeping mice are colored in red. These probes remain clustered and show systematic changes with variability as sleep progresses. **(B)**: The coefficient of variation of gene expression among mice deprived of sleep for 3, 6, 9 and 12 hours is plotted against the CV of the same gene at baseline. The probes with statistically significant changes in variability among sleeping mice are again colored red and now demonstrate a different temporal progression. **(C)**: The CV of probe expression among spontaneously sleeping mice after 3, 6, 9 and 12 is plotted against the CV of diurnally matched sleep deprived mice. The identified probes remain clustered at all circadian times and show systematic differences in inter-animal variability. Relative to baseline and sleep-deprivation, early sleep enhances inter-animal variability. At the end of the normal sleep period, animals allowed uninterrupted sleep show more uniform expression while sleep-deprived animals become relatively desynchronized. **(D)** The data from part **(C)** is re-plotted now highlighting all probes with Gene Ontology annotations related oxidation-reduction (n = 778, colored blue) and wound healing (n = 361, colored green). These large and physiologically important transcript groups show the same pattern and are synchronized after uninterrupted sleep.

Given the changes in lung clock-gene expression with sleep deprivation, we hypothesized that sleep and its associated changes in body temperature could function in synchronizing the peripheral circadian oscillators with behavioral rhythms. By extension, such changes would be expected to synchronize the peripheral oscillators of different animals with each other. Thus, we investigated the influence of sleep on the inter-animal variability of gene expression in the lung and questioned if the inter-animal variation of expression is a function of sleep duration. Bartlett’s test of homoscedasticity (uniform variance) [70,71] was applied to the five groups of experimental animals (baseline and sleeping for 3, 6, 9, and 12 hours) not subjected to artificial deprivation. The analysis yielded 1483 probes and 1122 unique named genes that showed changes in variability as a function of sleep duration (FDR <1%). Bartlett’s test is statistically powerful but sensitive to outliers and departures from normality. To safe-guard against this possibility we applied the more robust, but less powerful, Brown-Forsythe test [72] and retained only those transcripts that also showed significant (p < 0.01) changes in variability with this test. A total of 210 probes and 189 unique genes met these combined criteria for sleep/circadian related changes in inter-animal variability. We plotted (Figure 6A) the coefficient of variation (CV) of gene expression in each of the sleep groups as compared to its coefficient of variation at baseline. The genes identified as having statistically significant changes in variability were labeled red. Interestingly, these genes remain clustered and show the same temporal pattern in variability. The genes in the cluster become more variable during early sleep and more coherent at the end of the sleep period. Additional file 3: Figure S2 confirms that these changes in variability are not the result of sporadic outliers as the same pattern is observed with median absolute deviations. Further evidence that this group of genes is indeed a physiologically relevant cluster and not a random collection of genes is obtained from GO and KEGG-Pathway overrepresentation analysis (Table 5). This transcript cluster is *highly* enriched for annotations relating to oxidation/reduction, drug metabolism, and wound healing.

**Table 5:** Overrepresented functional annotations describing genes that show non-uniform variance as a function of sleep duration

**Annotation cluster 1**	**Enrichment score: 14.904263431729085**			
**Category**	**Term**	**P Value**	**Fold Enrichment**	**FDR**
SP_PIR_KEYWORDS	Microsome	4.15E-24	23.74	5.37E-21
KEGG_PATHWAY	Retinol metabolism	3.03E-20	17.72	3.19E-17
SP_PIR_KEYWORDS	Heme	3.00E-19	15.99	3.89E-16
SP_PIR_KEYWORDS	Monooxygenase	5.41E-19	19.11	7.00E-16
SP_PIR_KEYWORDS	Oxidoreductase	1.09E-18	6.50	1.41E-15
KEGG_PATHWAY	Drug metabolism	6.69E-18	15.30	7.05E-15
GOTERM_BP_FAT	GO:0055114 ~ oxidation reduction	3.07E-16	5.27	5.22E-13
SP_PIR_KEYWORDS	Iron	6.73E-15	8.04	8.76E-12
KEGG_PATHWAY	Metabolism of xenobiotics by cytochrome P450	7.52E-15	14.78	7.96E-12
KEGG_PATHWAY	Linoleic acid metabolism	3.42E-13	17.46	3.61E-10
SP_PIR_KEYWORDS	endoplasmic reticulum	4.12E-13	4.87	5.33E-10
KEGG_PATHWAY	Arachidonic acid metabolism	1.33E-07	8.30	1.40E-04
SP_PIR_KEYWORDS	metal-binding	4.05E-04	1.69	5.22E-01
**Annotation Cluster 2**	**Enrichment Score: 7.639984508416769**			
**Category**	**Term**	**P Value**	**Fold Enrichment**	**FDR**
KEGG_PATHWAY	Complement and coagulation cascades	3.36E-15	13.77	3.52E-12
GOTERM_BP_FAT	GO:0009611 ~ response to wounding	2.76E-10	5.96	4.32E-07
GOTERM_BP_FAT	GO:0007596 ~ blood coagulation	3.19E-08	14.07	4.99E-05
GOTERM_BP_FAT	GO:0050817 ~ coagulation	3.19E-08	14.07	4.99E-05
GOTERM_BP_FAT	GO:0007599 ~ hemostasis	3.63E-08	13.87	5.67E-05
GOTERM_BP_FAT	GO:0050878 ~ regulation of body fluid levels	2.66E-07	11.06	4.15E-04
SP_PIR_KEYWORDS	blood coagulation	1.33E-06	19.52	1.72E-03
GOTERM_BP_FAT	GO:0042060 ~ wound healing	1.89E-06	8.79	2.95E-03
SP_PIR_KEYWORDS	Plasma	7.60E-05	21.50	9.83E-02
**Annotation Cluster 3**	**Enrichment Score: 5.173741168245643**			
**Category**	**Term**	**P Value**	**Fold Enrichment**	**FDR**
KEGG_PATHWAY	Steroid hormone biosynthesis	2.85E-09	14.03	3.01E-06
KEGG_PATHWAY	Drug metabolism	5.61E-09	13.15	5.92E-06
KEGG_PATHWAY	Androgen and estrogen metabolism	1.09E-06	13.91	1.15E-03
KEGG_PATHWAY	Ascorbate and aldarate metabolism	3.62E-06	22.95	3.82E-03
KEGG_PATHWAY	Pentose and glucuronate interconversions	1.67E-04	16.88	1.76E-01
KEGG_PATHWAY	mmu00500:Starch and sucrose metabolism	3.41E-04	9.56	3.59E-01
SP_PIR_KEYWORDS	Glycosyltransferase	7.02E-04	4.67	9.04E-01

Annotation clusters were provided by DAVID. Only annotations with an FDR <5% are retained and only the top 3 clusters are shown.

As the five experimental groups considered in this analysis differed not only in sleep duration but also in circadian time, we next plotted the CVs among the four sleep-deprived groups as compared to baseline (Figure 6B). The same transcripts are labeled red. Despite being identified from the analysis of an independent group of animals under different conditions, these genes remain a single cluster but demonstrate a different temporal progression during sleep deprivation. Among sleep-deprived animals, the genes exhibit increased coherence during early sleep deprivation but later return to, and then exceed baseline variability. Thus, the inter-animal variability of this gene cluster changes systematically with both circadian time and sleep/wake behavioral state. When the coefficient of variation in gene expression among sleeping animals is compared to the coefficient of variation among diurnally matched sleep-deprived animals (Figure 6C), this pattern becomes clear. The cluster of genes rotates in a counter clockwise direction as the animals allowed undisturbed sleep show synchronization during the later sleep period, while sleep deprived animals sacrificed at the same diurnal time show increasing inter-animal variability. Finally (Figure 6D) we followed all 778 probes that mapped to Gene Ontology (GO) annotations “GO:0055114 ~ oxidation reduction” and all 361 probes that mapped to “GO:0009611 ~ response to wounding”, both of which were highly enriched in the transcript cluster. These entire gene groups demonstrate the same systematic changes in transcript variability with sleep and sleep deprivation.

The same classes of transcripts that were synchronized by sleep have been identified among the most enriched annotations describing circadian transcripts in the rat lung [73]. The ability for sleep to act as a synchronizing cue for peripheral oscillators suggests that sleep may have a role in coordinating the physiology of peripheral tissues and matching that physiology to behavioral rhythms. We hypothesize that sleep itself, or the extended periods of altered temperature and neurohumoral tone associated with sleep, can act as a “reset button” for peripheral tissues. Moreover, this effect is likely to be most pronounced in tissues, like the lung, with poor intercellular communication and less robust rhythms [74]. As a result, the metabolic consequences of sleep deprivation on peripheral tissues may be intimately connected to the effects of chronic circadian disruption and jet lag with. Without the synchronizing effects of sleep, peripheral tissues are apt to become progressively out-of-sync and poorly coordinated with behavior.

## Conclusions

Taken together, these results show that the transcriptional effects of sleep extend beyond the brain to include peripheral tissues. The physiological roles of the heart and lung show a stereotyped consistency between sleep and wake. Yet, these tissues show considerable transcriptional changes with sleep/wake behavioral state. The transcriptional response to sleep and wake in peripheral tissues shows a marked similarity to that observed in brain. In the heart and lung this similarity in the response to sleep extends beyond the names of the genes and pathways that are regulated and includes a similar temporal pattern of activation. Sleep reduces expression of genes involved in cellular stress and enhances the transcription of reducing agents. These results are generally consistent with transcriptional changes observed in the brain across various model species. In contrast to the commonality of gene pathways repressed by sleep, different molecular processes were enhanced by sleep in heart, lung, and brain. In each case sleep seems to provide a temporal compartment to cope with the tissue-specific molecular consequences of wakefulness.

A fundamental limitation of our study is that it does not partition which of these effects are due to physical rest and inactivity as opposed to true sleep. While similar criticisms could be leveled against previous transcriptional findings in the brain, there is plentiful evidence that some elements of cognitive recovery after sleep deprivation specifically require sleep rather than physical inactivity. Further research will be needed to elucidate which of the transcriptional consequences ascribed to sleep and sleep deprivation are more general results of physical rest.

As has been noted before, animals in the undisturbed sleep group were active during a portion of the sleep period while it is very likely that some of the sleep-deprived animals did get short periods of sleep. Given the lack of EEG monitoring we cannot quantify the extent of this limitation. While we identify a significant fraction of the heart and lung transcriptomes as being influenced by sleep, the presence of sleep in the sleep-deprived group may have reduced the sensitivity of the experiment in identifying sleep responsive transcriptional changes. It is also worth noting that while the restricted feeding paradigm used in this study ensures that the sleep deprived and undisturbed animals did not have a differential food intake during the study, we cannot exclude that a physiologically important interaction between dietary restriction and sleep deprivation may contribute to our results.

Finally, our data show that sleep or behavioral activity may play a role in synchronizing the peripheral transcription of a group of physiologically important transcripts in the lung. This finding requires further exploration, but suggests a unique role for the function of sleep in peripheral tissues. Indeed, a recent study in humans concluded that the chronic sleep deprivation blunts circadian oscillations in blood [75]. Our data are limited to a 12 hour window of acute deprivation and thus do not permit the assessment of circadian amplitude. However, the finding that sleep synchronizes cellular oscillations and influences *Cibp* expression may help explain that result. Thus, this data adds to the growing evidence that the functions of sleep extend beyond the brain and also include the modulation of peripheral circadian rhythms.

## Methods

Mice were housed under conditions meeting the guidelines issued by the Association for Assessment and Accreditation of Laboratory Animal Care. All animal protocols were approved by the University of Pennsylvania’s Institutional Animal Care and Use Committee (IACUC protocol #801832).

### Animal processing

Experiments were performed on male mice (C57BL/6 J) 10 +/−1 wk of age. Mice were kept in a pathogen-free environment with 12 hr:12 hr light:dark cycle. Temperature and humidity were regulated (22°C and 45–55% respectively). Water was available *ad libitum*. Food was accessible only during the 12 hours of the active period. Animals were acclimated to this feeding pattern for 14 days prior to the beginning of the deprivation and tissue collection protocols. This was done to avoid differential food intake between mice that were subsequently sleep deprived during the lights-on period and those allowed uninterrupted sleep. This acclimatization period was also used to allow animals to become accustomed to the extended presence of an experimenter and repeated gentle touching. This likely reduced, but did not fully remove stress resulting from the subsequent sleep deprivation procedure. Mice were sacrificed by cervical dislocation following 3, 6, 9, and 12 h of total sleep deprivation (*n* = 8 or 9 at each time point). Deprivation was initiated at lights-on and performed through gentle handling. Mice that had been allowed undisturbed sleep were sacrificed at the same diurnal time points as sleep-deprived mice. An additional baseline group (*n* = 10) was sacrificed at time zero, i.e., at the time of lights-on at 7:00 AM. All mice were behaviorally monitored with the AccuScan infrared monitoring system that detects movement when the mouse crosses electronic beams (Columbus Instruments). For each animal, descriptive statistics such as average activity (beam breaks) for each hour (and then averaged across 12 h of light and dark) and estimated sleep amounts were computed. Sleep was considered to be present when there was 40 s or more of continuous inactivity. This has been shown previously to provide accurate estimates of sleep and wake [76]. In addition, plots of activity and sleep graphed as moving averages broken down by light/dark cycle per day were generated for visual inspection. All mice studied had normal sleep/wake behavior for this strain at this age.

### Tissue sampling, RNA isolation, and microarrays

The animals were subjected to cervical dislocation followed by tissue collection. Total RNA was isolated with TRIzol (Invitrogen-Life, Carlsbad, CA) and further purified using RNeasy columns (Qiagen, Valencia, CA) as recommended by the manufacturer’s instructions. Transcript levels were assayed using the GeneChip® Mouse Gene 1.0 ST array (Affymetrix, Santa Clara, CA), comprising 28.000 genes. RNA quality and integrity was checked using spectrophotometer to access the concentration and purity in ratio of absorbance at 260 nm. A total of 100 ng of RNA from each time point from each animal and respectively tissue was used to hybridize independents sets of Gene Chip Mouse Gene St 1.0 arrays (Affymetrix®, Santa Clara, CA, USA), according to the manufacturer’s protocol and following the best practice and high-standard criteria required. The RNA processing and microarrays experimental protocols were performed in the University of Pennsylvania Microarray Core Facility. Each sample was analyzed with a single microarray.

Target preparation, including hybridization and post-hybridization procedures, was performed as described by the Affymetrix GeneChip Whole-transcriptome Terminal Labeling and Controls manual (http://www.affymetrix.com). Each of the 28,853 genes is represented on the array by approximately 27 probes spread across the full length of the gene.

### Preprocessing and data analysis

Probe intensity data from all arrays were read into the R software environment (http://www.R-project.org) directly from .cel files using the R/oligo package [77,78]. R/oligo was also used to extract probe level data to assess data quality using intensity histograms and boxplots and to create summary measures of expression. Normalization was performed using the robust multi-array average (RMA) method to form one expression measure for each gene on each array histograms [79]. RMA processing was performed separately for each tissue (heart, lung), using all data sets from a given tissue together. Data analysis proceeded in a staged approach. The first step was an analysis of variance (ANOVA)-based approach to find gene expression differences between behavioral states (sleep or sleep deprived). The data from the animals at the baseline group (time zero, lights on) were not used in this first step of the analysis. Overall contrasts across conditions were considered by implementing gene-specific, fixed-effect ANOVA models [80] using the R/maanova package [81]. Specifically, the model Y_i_ = μ + STATE + e_i_ was used to fit gene expression measures within each tissue, where μ is the mean for each array, STATE is the effect for each behavioral state (sleep deprived or sleeping), and e_i_ captures random error. The first analysis looked for a main effect of state, comparing expression levels between all sleep-deprived (awake) and sleeping (sleep) animals. All statistical tests were conducted with a modified F-statistic (Fs) that that shrinks variance components based on information from all the probe sets on the array [82]. Critical *P* values were calculated through permutation analyses incorporating 1,000 sample shuffles and pooled *F*-statistics [83]. False discovery rate (FDR) values were determined using the Benjamini Hochberg method [84] implemented through the p. adjust function in R. An FDR threshold of 1% was used to determine differential gene expression.

Transcripts differentially expressed between sleep/wake behavioral states (step 1) were included in a secondary analysis to test for temporal expression changes among the experimental and baseline animals not subjected to gentle handling and enforced sleep deprivation. This secondary trend analysis utilized the data obtained from both mice sacrificed at lights-on (time zero at 7:00 AM) and the animals sacrificed after different durations of spontaneous sleep (i.e., at 10:00 AM, 1:00 PM, 4:00 PM, 7:00 PM). Trend tests were performed through linear regression analyses in which time was treated as a continuous variable. For each tissue, expression measures were fit to the model Y_i(t)_ = α_0(i)_ + ß_j(i)_t + ϵ_ijk_, which included common intercepts (α_0_) and unique slopes over time (ß_j_) for each state (j = 1,2 for sleep and sleep deprivation, respectively). The temporal pattern of differential gene expression over time was determined according to the slope (positive or negative). Statistical significance was determined as described in step 1. Strict sleep enhanced (sleep repressed) genes met criteria for differential expression in the first analysis and showed a significantly positive (or negative) slope during the spontaneous sleep period (FDR <1%). Unless otherwise noted all other plots and data analysis were done in the R programming environment.

### Clustering and heatmap generation

For each gene found to have sleep dependant transcription (either sleep repressed or sleep enhanced) in both the heart and lung, we created an eight dimensional vector describing the difference in mean mRNA expression between the sleep and sleep deprived cohorts as a function of time (mean differences in heart and lung at times 3, 6, 9, and12). The agglomerative clustering command (Agglomerate) in Mathematica v. 8.0 (Wolfram Research) was used with the Euclidean distance metric to provide a relative ordering of the transcript expression vectors. The heat map was generated with the ArrayPlot command. Clustering was only done to order the genes for vertical placement on the resulting heat map.

### DAVID analysis

We used Database for Annotation, Visualization and Integrated Discovery (DAVID) [28,29] bioinformatics toolbox to identify significant overrepresentation in one of the following categories: gene ontology (GO) biological process, molecular function, and Protein Information Resource (PIR) Key words, Kyoto Encyclopedia of Genes and Genomes (KEGG) pathways. Over-represented functional categories are identified by calculation of a conservative adjustment to the one-tailed Fisher’s exact probability that represents the upper bound of the distribution. In addition, we used the within-system FDR (False discovery rate) to assess the impact of multiple testing using an FDR cutoff of 5%.

To aid in interpreting these results the functional clustering tool available within DAVID was used to group overrepresented biological terms that represent overlapping gene sets and common physiology. The clustering stringency was set to “medium”. Tables were constructed from DAVID clustering output by maintaining the clusters but including only the enriched terms that met the FDR threshold.

### KEGG pathway visualization

To generate figures of the imputed pathways, the lists of sleep specific transcripts were loaded onto the KEGG Mapper search and color tool [85] (http://www.genome.jp/kegg/tool/map_pathway2.html). The protein processing pathway was redrawn to enhance resolution.

### Uniformity of variance analysis

Only data from animals sacrificed at baseline and after uninterrupted sleep was used in this analysis. Log base 2 expression intensities for each probe were grouped based on the duration of uninterrupted sleep (baseline-0, 3 hrs, 6 hrs, 9 hrs, and 12 hrs). Bartlett’s test of homoscedasticity [70] was used to assess the homogeneity of variance among all 5 groups. Testing was done in the R programming environment using the *bartlett.test()* function from library *stats*. Resulting p values were corrected for multiple testing by the method of Benjamini and Hochberg [84] with the *p.adjust()* command. All probes with a resulting q value <0.01 were retained for further analysis. The implementation of the Brown-Forsythe [72] test in R package *lawstat* was used as a confirmatory nonparametric test. Probes that passed secondary screening with a p value <0.01 were considered to have significant changes in variance as a function of sleep duration and/or diurnal time.

## Availability of supporting data

The data set supporting the results of this article are available at the N.I.H. Gene Expression Omnibus (GEO) accession # GSE42323 (http://www.ncbi.nlm.nih.gov/geo/).

## Competing interests

The authors declare that they have no competing interests.

## Authors’ contributions

RA analyzed the data, performed bioinformatics analysis, and drafted the manuscript. RP co-wrote the manuscript, collaborated in bioinformatics analysis, and participated in sample collection. KS performed statistical analysis and assisted in writing the manuscript. MR collected and processed samples and contributed to data analysis. ST contributed in study design and drafted the manuscript. AP oversaw the study design, data interpretation, and drafted the manuscript. All authors read and approved the final version of the manuscript.

## Supplementary Material

Additional file 1 Comma separated value (CSV) file listing sleep enhanced and sleep repressed genes in heart and lung. Click here for file

Additional file 2: Figure S1 KEGG Pathway diagram depicting ubiquitin mediated proteolysis. Transcripts enhanced by sleep in the heart are colored light blue. Transcripts enhanced by sleep in both heart and lungs are colored dark blue. Click here for file

Additional file 3: Figure S2 Effect of sleep and sleep deprivation on the inter-animal variability of transcript expression in the lung as assessed by median deviations. 210 probes showed statistically significant changes in expression variability as a function of sleep duration. The median absolute deviation (MAD) is divided by median expression to obtain a normalized measure of variation resistant to outliers. The normalized MAD among spontaneously sleeping mice after 3, 6, 9 and 12 hours is plotted against the normalized MAD of the same transcript at baseline (lights-on). The red line of identity (x = y) demarcates no change in inter-animal variability. Transcripts with statistically significant temporal changes in variance among sleeping mice were colored in red. As shown in Figure 6A, these transcripts remain clustered and show systematic changes with variability as sleep progresses. Click here for file

## References

[B1] Cirelli C Tononi G Is sleep essential? PLoS Biol 2008 6 e216 10.1371/journal.pbio.0060216 18752355 PMC2525690

[B2] Tononi G Cirelli C Sleep and synaptic homeostasis: a hypothesis Brain Res Bull 2003 62 143 150 10.1016/j.brainresbull.2003.09.004 14638388

[B3] Benington JH Heller HC Restoration of brain energy metabolism as the function of sleep Prog Neurobiol 1995 45 347 360 10.1016/0301-0082(94)00057-O 7624482

[B4] Scharf MT Naidoo N Zimmerman JE Pack AI The energy hypothesis of sleep revisited Prog Neurobiol 2008 86 264 280 10.1016/j.pneurobio.2008.08.003 18809461 PMC2948963

[B5] Walker MP Stickgold R Sleep, memory, and plasticity Annu Rev Psychol 2006 57 139 166 10.1146/annurev.psych.56.091103.070307 16318592

[B6] Mackiewicz M Shockley KR Romer MA Galante RJ Zimmerman JE Naidoo N Baldwin DA Jensen ST Churchill GA Pack AI Macromolecule biosynthesis: a key function of sleep Physiol Genomics 2007 31 441 457 10.1152/physiolgenomics.00275.2006 17698924

[B7] Hobson JA Sleep is of the brain, by the brain and for the brain Nature 2005 437 1254 1256 10.1038/nature04283 16251949

[B8] Rechtschaffen A Gilliland MA Bergmann BM Winter JB Physiological correlates of prolonged sleep deprivation in rats Science (New York, N.Y.) 1983 221 182 184 10.1126/science.6857280 6857280

[B9] Buxton OM Cain SW O’Connor SP Porter JH Duffy JF Wang W Czeisler CA Shea SA Adverse metabolic consequences in humans of prolonged sleep restriction combined with circadian disruption Sci Transl Med 2012 4 129ra43 10.1126/scitranslmed.3003200 22496545 PMC3678519

[B10] Van Cauter E Spiegel K Tasali E Leproult R Metabolic consequences of sleep and sleep loss Sleep Med 2008 9 Suppl 1 S23 S28 18929315 10.1016/S1389-9457(08)70013-3 PMC4444051

[B11] Hanlon EC Van Cauter E Quantification of sleep behavior and of its impact on the cross-talk between the brain and peripheral metabolism Proc Natl Acad Sci USA 2011 108 15609 15616 10.1073/pnas.1101338108 21852576 PMC3176603

[B12] Faraut B Boudjeltia KZ Vanhamme L Kerkhofs M Immune, inflammatory and cardiovascular consequences of sleep restriction and recovery Sleep Med Rev 2012 16 137 149 10.1016/j.smrv.2011.05.001 21835655

[B13] Lange T Dimitrov S Born J Effects of sleep and circadian rhythm on the human immune system Ann N Y Acad Sci 2010 1193 48 59 10.1111/j.1749-6632.2009.05300.x 20398008

[B14] Mackiewicz M Zimmerman JE Shockley KR Churchill GA Pack AI What are microarrays teaching us about sleep? Trends Mol Med 2009 15 79 87 10.1016/j.molmed.2008.12.002 19162550 PMC2942088

[B15] Cirelli C Gutierrez CM Tononi G Extensive and divergent effects of sleep and wakefulness on brain gene expression Neuron 2004 41 35 43 10.1016/S0896-6273(03)00814-6 14715133

[B16] Maret S Dorsaz S Gurcel L Pradervand S Petit B Pfister C Hagenbuchle O O’Hara BF Franken P Tafti M Homer1a is a core brain molecular correlate of sleep loss Proc Natl Acad Sci USA 2007 104 20090 20095 10.1073/pnas.0710131104 18077435 PMC2148427

[B17] Zimmerman JE Rizzo W Shockley KR Raizen DM Naidoo N Mackiewicz M Churchill GA Pack AI Multiple mechanisms limit the duration of wakefulness in Drosophila brain Physiol Genomics 2006 27 337 350 10.1152/physiolgenomics.00030.2006 16954408

[B18] Cirelli C LaVaute TM Tononi G Sleep and wakefulness modulate gene expression in Drosophila J Neurochem 2005 94 1411 1419 10.1111/j.1471-4159.2005.03291.x 16001966

[B19] Jones S Pfister-Genskow M Benca RM Cirelli C Molecular correlates of sleep and wakefulness in the brain of the white-crowned sparrow J Neurochem 2008 105 46 62 10.1111/j.1471-4159.2007.05089.x 18028333

[B20] Wang H Liu Y Briesemann M Yan J Computational analysis of gene regulation in animal sleep deprivation Physiol Genomics 2010 42 427 436 10.1152/physiolgenomics.00205.2009 20501693

[B21] Naidoo N Cellular stress/the unfolded protein response: relevance to sleep and sleep disorders Sleep Med Rev 2009 13 195 204 10.1016/j.smrv.2009.01.001 19329340 PMC2964262

[B22] Naidoo N Giang W Galante RJ Pack AI Sleep deprivation induces the unfolded protein response in mouse cerebral cortex J Neurochem 2005 92 1150 1157 10.1111/j.1471-4159.2004.02952.x 15715665

[B23] Lange T Perras B Fehm HL Born J Sleep enhances the human antibody response to hepatitis A vaccination Psychosom Med 2003 65 831 835 10.1097/01.PSY.0000091382.61178.F1 14508028

[B24] Nagai M Hoshide S Kario K Sleep duration as a risk factor for cardiovascular disease- a review of the recent literature Curr Cardiol Rev 2010 6 54 61 10.2174/157340310790231635 21286279 PMC2845795

[B25] Broussard JL Ehrmann DA Van Cauter E Tasali E Brady MJ Impaired insulin signaling in human adipocytes after experimental sleep restriction: a randomized, crossover study Ann Intern Med 2012 157 549 557 10.7326/0003-4819-157-8-201210160-00005 23070488 PMC4435718

[B26] Spiegel K Leproult R Van Cauter E Impact of sleep debt on metabolic and endocrine function Lancet 1999 354 1435 1439 10.1016/S0140-6736(99)01376-8 10543671

[B27] Dattilo M Antunes HKM Medeiros A Mônico-Neto M De Souza HS Lee KS Tufik S De Mello MT Paradoxical sleep deprivation induces muscle atrophy Muscle Nerve 2012 45 431 433 10.1002/mus.22322 22334180

[B28] Huang DW Sherman BT Lempicki RA Systematic and integrative analysis of large gene lists using DAVID bioinformatics resources Nat Protoc 2009 4 44 57 19131956 10.1038/nprot.2008.211

[B29] Huang DW Sherman BT Lempicki RA Bioinformatics enrichment tools: paths toward the comprehensive functional analysis of large gene lists Nucleic Acids Res 2009 37 1 13 10.1093/nar/gkn923 19033363 PMC2615629

[B30] Shaw PJ Correlates of Sleep and Waking in Drosophila melanogaster Science 2000 287 1834 1837 10.1126/science.287.5459.1834 10710313

[B31] Mongrain V Hernandez SA Pradervand S Dorsaz S Curie T Hagiwara G Gip P Heller HC Franken P Separating the contribution of glucocorticoids and wakefulness to the molecular and electrophysiological correlates of sleep homeostasis Sleep 2010 33 1147 1157 20857860 10.1093/sleep/33.9.1147 PMC2938796

[B32] Cirelli C Faraguna U Tononi G Changes in brain gene expression after long-term sleep deprivation J Neurochem 2006 98 1632 1645 10.1111/j.1471-4159.2006.04058.x 16923172

[B33] Schröder M Kaufman RJ The mammalian unfolded protein response Annu Rev Biochem 2005 74 739 789 10.1146/annurev.biochem.73.011303.074134 15952902

[B34] Ron D Walter P Signal integration in the endoplasmic reticulum unfolded protein response Nat Rev Mol Cell Biol 2007 8 519 529 10.1038/nrm2199 17565364

[B35] Sugden MC Holness MJ Recent advances in mechanisms regulating glucose oxidation at the level of the pyruvate dehydrogenase complex by PDKs Am J Physiol Endocrinol Metab 2003 284 E855 E862 12676647 10.1152/ajpendo.00526.2002

[B36] Eaton DL Bammler TK Concise review of the glutathione S-transferases and their significance to toxicology Toxicological sciences: an official journal of the Society of Toxicology 1999 49 156 164 10.1093/toxsci/49.2.156 10416260

[B37] Meyer Y Buchanan BB Vignols F Reichheld J-P Thioredoxins and glutaredoxins: unifying elements in redox biology Annu Rev Genet 2009 43 335 367 10.1146/annurev-genet-102108-134201 19691428

[B38] Kimura M Kapás L Krueger JM Oxidized glutathione promotes sleep in rabbits Brain Res Bull 1998 45 545 548 10.1016/S0361-9230(97)00441-3 9566496

[B39] D’Almeida V Lobo LL Hipólide DC De Oliveira AC Nobrega JN Tufik S Sleep deprivation induces brain region-specific decreases in glutathione levels NeuroReport 1998 9 2853 2856 10.1097/00001756-199808240-00031 9760133

[B40] Everson CA Laatsch CD Hogg N Antioxidant defense responses to sleep loss and sleep recovery Am J Physiol Regul Integr Comp Physiol 2005 288 R374 R383 15472007 10.1152/ajpregu.00565.2004

[B41] Ramanathan L Hu S Frautschy SA Siegel JM Short-term total sleep deprivation in the rat increases antioxidant responses in multiple brain regions without impairing spontaneous alternation behavior Behav Brain Res 2010 207 305 309 10.1016/j.bbr.2009.10.014 19850085 PMC2815069

[B42] Rahman I Oxidative stress, chromatin remodeling and gene transcription in inflammation and chronic lung diseases J Biochem Mol Biol 2003 36 95 109 10.5483/BMBRep.2003.36.1.095 12542980

[B43] Smyth HS Sleight P PICKERING GW Reflex Regulation of Arterial Pressure during Sleep in Man: A Quantitative Method of Assessing Baroreflex Sensitivity Circ Res 1969 24 109 121 10.1161/01.RES.24.1.109 4303309

[B44] Monti A Medigue C Nedelcoux H Escourrou P Autonomic control of the cardiovascular system during sleep in normal subjects Eur J Appl Physiol 2002 87 174 181 10.1007/s00421-002-0597-1 12070629

[B45] Lecker SH Solomon V Mitch WE Goldberg AL Muscle protein breakdown and the critical role of the ubiquitin-proteasome pathway in normal and disease states J Nutr 1999 129 227S 237S 9915905 10.1093/jn/129.1.227S

[B46] Kipreos ET Pagano M The F-box protein family Genome Biol 2000 1 5 1 REVIEWS3002 11178263 10.1186/gb-2000-1-5-reviews3002 PMC138887

[B47] Hirsch C Gauss R Horn SC Neuber O Sommer T The ubiquitylation machinery of the endoplasmic reticulum Nature 2009 458 453 460 10.1038/nature07962 19325625

[B48] Hall JE Guyton and Hall Textbook of Medical Physiology: Enhanced E-book 2010 Philadelphia: Elsevier Health Sciences 1120

[B49] Bergmann O Bhardwaj RD Bernard S Zdunek S Barnabé-Heider F Walsh S Zupicich J Alkass K Buchholz BA Druid H Jovinge S Frisén J Evidence for cardiomyocyte renewal in humans Science (New York, N.Y.) 2009 324 98 102 10.1126/science.1164680 PMC2991140 19342590

[B50] Cesselli D Jakoniuk I Barlucchi L Beltrami AP Hintze TH Nadal-Ginard B Kajstura J Leri A Anversa P Oxidative stress-mediated cardiac cell death is a major determinant of ventricular dysfunction and failure in dog dilated cardiomyopathy Circ Res 2001 89 279 286 10.1161/hh1501.094115 11485979

[B51] Hare JM Oxidative Stress and Apoptosis in Heart Failure Progression Circ Res 2001 89 198 200 11485969

[B52] Siggens L Figg N Bennett M Foo R Nutrient deprivation regulates DNA damage repair in cardiomyocytes via loss of the base-excision repair enzyme OGG1 FASEB journal: official publication of the Federation of American Societies for Experimental Biology 2012 26 2117 2124 10.1096/fj.11-197525 22302830 PMC3630495

[B53] Singh KK Shukla PC Quan A Desjardins J-F Lovren F Pan Y Garg V Gosal S Garg A Szmitko PE Schneider MD Parker TG Stanford WL Leong-Poi H Teoh H Al-Omran M Verma S BRCA2 protein deficiency exaggerates doxorubicin-induced cardiomyocyte apoptosis and cardiac failure J Biol Chem 2012 287 6604 6614 10.1074/jbc.M111.292664 22157755 PMC3325595

[B54] Kang T-H Reardon JT Kemp M Sancar A Circadian oscillation of nucleotide excision repair in mammalian brain Proc Natl Acad Sci USA 2009 106 2864 2867 10.1073/pnas.0812638106 19164551 PMC2629438

[B55] Kang T-H Lindsey-Boltz LA Reardon JT Sancar A Circadian control of XPA and excision repair of cisplatin-DNA damage by cryptochrome and HERC2 ubiquitin ligase Proc Natl Acad Sci USA 2010 107 4890 4895 10.1073/pnas.0915085107 20304803 PMC2841896

[B56] Franken P Dijk D-J Circadian clock genes and sleep homeostasis Eur J Neurosci 2009 29 1820 1829 10.1111/j.1460-9568.2009.06723.x 19473235

[B57] Franken P Dudley CA Estill SJ Barakat M Thomason R O’Hara BF McKnight SL NPAS2 as a transcriptional regulator of non-rapid eye movement sleep: genotype and sex interactions Proc Natl Acad Sci USA 2006 103 7118 7123 10.1073/pnas.0602006103 16636276 PMC1459027

[B58] Wisor JP Pasumarthi RK Gerashchenko D Thompson CL Pathak S Sancar A Franken P Lein ES Kilduff TS Sleep deprivation effects on circadian clock gene expression in the cerebral cortex parallel electroencephalographic differences among mouse strains The Journal of neuroscience: the official journal of the Society for Neuroscience 2008 28 7193 7201 10.1523/JNEUROSCI.1150-08.2008 18614689 PMC2603080

[B59] Honma S Kawamoto T Takagi Y Fujimoto K Sato F Noshiro M Kato Y Honma K Dec1 and Dec2 are regulators of the mammalian molecular clock Nature 2002 419 841 844 10.1038/nature01123 12397359

[B60] He Y Jones CR Fujiki N Xu Y Guo B Holder JL Rossner MJ Nishino S Fu Y-H The transcriptional repressor DEC2 regulates sleep length in mammals Science (New York, N.Y.) 2009 325 866 870 10.1126/science.1174443 PMC2884988 19679812

[B61] Saini C Morf J Stratmann M Gos P Schibler U Simulated body temperature rhythms reveal the phase-shifting behavior and plasticity of mammalian circadian oscillators Genes Dev 2012 26 567 580 10.1101/gad.183251.111 22379191 PMC3315118

[B62] Buhr ED Yoo S-H Takahashi JS Science (New York, N.Y.) 2010 330 379 385 10.1126/science.1195262 PMC3625727 20947768

[B63] Brown SA Zumbrunn G Fleury-Olela F Preitner N Schibler U Rhythms of mammalian body temperature can sustain peripheral circadian clocks Current biology: CB 2002 12 1574 1583 10.1016/S0960-9822(02)01145-4 12372249

[B64] Morf J Rey G Schneider K Stratmann M Fujita J Naef F Schibler U Cold-inducible RNA-binding protein modulates circadian gene expression posttranscriptionally Science (New York, N.Y.) 2012 338 379 383 10.1126/science.1217726 22923437

[B65] Dardente H Mendoza J Fustin J-M Challet E Hazlerigg DG Implication of the F-Box Protein FBXL21 in circadian pacemaker function in mammals PLoS One 2008 3 e3530 10.1371/journal.pone.0003530 18953409 PMC2568807

[B66] Hirano A Yumimoto K Tsunematsu R Matsumoto M Oyama M Kozuka-Hata H Nakagawa T Lanjakornsiripan D Nakayama KI Fukada Y FBXL21 Regulates Oscillation of the Circadian Clock through Ubiquitination and Stabilization of Cryptochromes Cell 2013 152 1106 1118 10.1016/j.cell.2013.01.054 23452856

[B67] Yoo S-H Mohawk JA Siepka SM Shan Y Huh SK Hong H-K Kornblum I Kumar V Koike N Xu M Nussbaum J Liu X Chen Z Chen ZJ Green CB Takahashi JS Competing E3 Ubiquitin Ligases Govern Circadian Periodicity by Degradation of CRY in Nucleus and Cytoplasm Cell 2013 152 1091 1105 10.1016/j.cell.2013.01.055 23452855 PMC3694781

[B68] Fonjallaz P Ossipow V Wanner G Schibler U The two PAR leucine zipper proteins, TEF and DBP, display similar circadian and tissue-specific expression, but have different target promoter preferences EMBO J 1996 15 351 362 8617210 PMC449950

[B69] Mongrain V La Spada F Curie T Franken P Sleep loss reduces the DNA-binding of BMAL1, CLOCK, and NPAS2 to specific clock genes in the mouse cerebral cortex PLoS One 2011 6 e26622 10.1371/journal.pone.0026622 22039518 PMC3200344

[B70] Bartlett M Properties of sufficiency and statistical tests Proceedings of the Royal Society of London. Series A, Mathematical and Physical Sciences 1937 160 268 282 10.1098/rspa.1937.0109

[B71] NIST/SEMATECH e-Handbook of Statistical Methods[http://www.itl.nist.gov/div898/handbook

[B72] Brown MB Forsythe AB Robust Tests for the Equality of Variances J Am Stat Assoc 1974 69 364 367 10.1080/01621459.1974.10482955

[B73] Sukumaran S Jusko WJ Dubois DC Almon RR Light–dark oscillations in the lung transcriptome: implications for lung homeostasis, repair, metabolism, disease, and drug action J Appl Physiol 2011 110 1732 1747 Bethesda, Md. : 1985 10.1152/japplphysiol.00079.2011 21436464 PMC3119131

[B74] Abraham U Granada AE Westermark PO Heine M Kramer A Herzel H Coupling governs entrainment range of circadian clocks Mol Syst Biol 2010 6 438 21119632 10.1038/msb.2010.92 PMC3010105

[B75] Möller-Levet CS Archer SN Bucca G Laing EE Slak A Kabiljo R Lo JCY Santhi N Von Schantz M Smith CP Dijk D-J Effects of insufficient sleep on circadian rhythmicity and expression amplitude of the human blood transcriptome Proceedings of the National Academy of Sciences of the United States of America 2013 110 12 E1132 41 10.1073/pnas.1217154110 23440187 PMC3607048

[B76] Pack AI Galante RJ Maislin G Cater J Metaxas D Lu S Zhang L Von Smith R Kay T Lian J Svenson K Peters LL Novel method for high-throughput phenotyping of sleep in mice Physiol Genomics 2007 28 232 238 16985007 10.1152/physiolgenomics.00139.2006

[B77] Carvalho BS Irizarry RA A framework for oligonucleotide microarray preprocessing Bioinformatics (Oxford, England) 2010 26 2363 7 10.1093/bioinformatics/btq431 PMC2944196 20688976

[B78] Carvalho B Bengtsson H Speed TP Irizarry RA Exploration, normalization, and genotype calls of high-density oligonucleotide SNP array data Biostatistics (Oxford, England) 2007 8 485 499 10.1093/biostatistics/kxl042 17189563

[B79] Irizarry RA Hobbs B Collin F Beazer-Barclay YD Antonellis KJ Scherf U Speed TP Exploration, normalization, and summaries of high density oligonucleotide array probe level data Biostatistics (Oxford, England) 4 249 264 10.1093/biostatistics/4.2.249 12925520

[B80] Churchill GA Using ANOVA to analyze microarray data Biotechniques 2004 37 177 173 175 15335204 10.2144/04372TE01

[B81] Wu H Kerr M Cui X Churchill G MAANOVA Parmigiani G, Garrett E, Irizarry R, Zeger S A Software Package for the Analysis of Spotted cDNA Microarray Experiments The Analysis of Gene Expression Data 2003 New York: Springer-Verlag 313 341

[B82] Cui X Hwang JTG Qiu J Blades NJ Churchill GA Improved statistical tests for differential gene expression by shrinking variance components estimates Biostatistics (Oxford, England) 2005 6 59 75 10.1093/biostatistics/kxh018 15618528

[B83] Yang H Churchill G Estimating p-values in small microarray experiments Bioinformatics (Oxford, England) 2007 23 38 43 10.1093/bioinformatics/btl548 17077100

[B84] Benjamini Y Hochberg Y Controlling the false discovery rate: a practical and powerful approach to multiple testing Journal of the Royal Statistical Society, Series B 1995 57 1 289 300

[B85] Kanehisa M Goto S Furumichi M Tanabe M Hirakawa M KEGG for representation and analysis of molecular networks involving diseases and drugs Nucleic Acids Res 2010 38 D355 D360 10.1093/nar/gkp896 19880382 PMC2808910

