# Supplementary figures and images for: Sleep is not just for the brain: transcriptional responses to sleep in peripheral tissues
Source: BMC Genomics. 2013 May 30;14:362. doi: 10.1186/1471-2164-14-362 (PMC3701596; doi:10.1186/1471-2164-14-362)

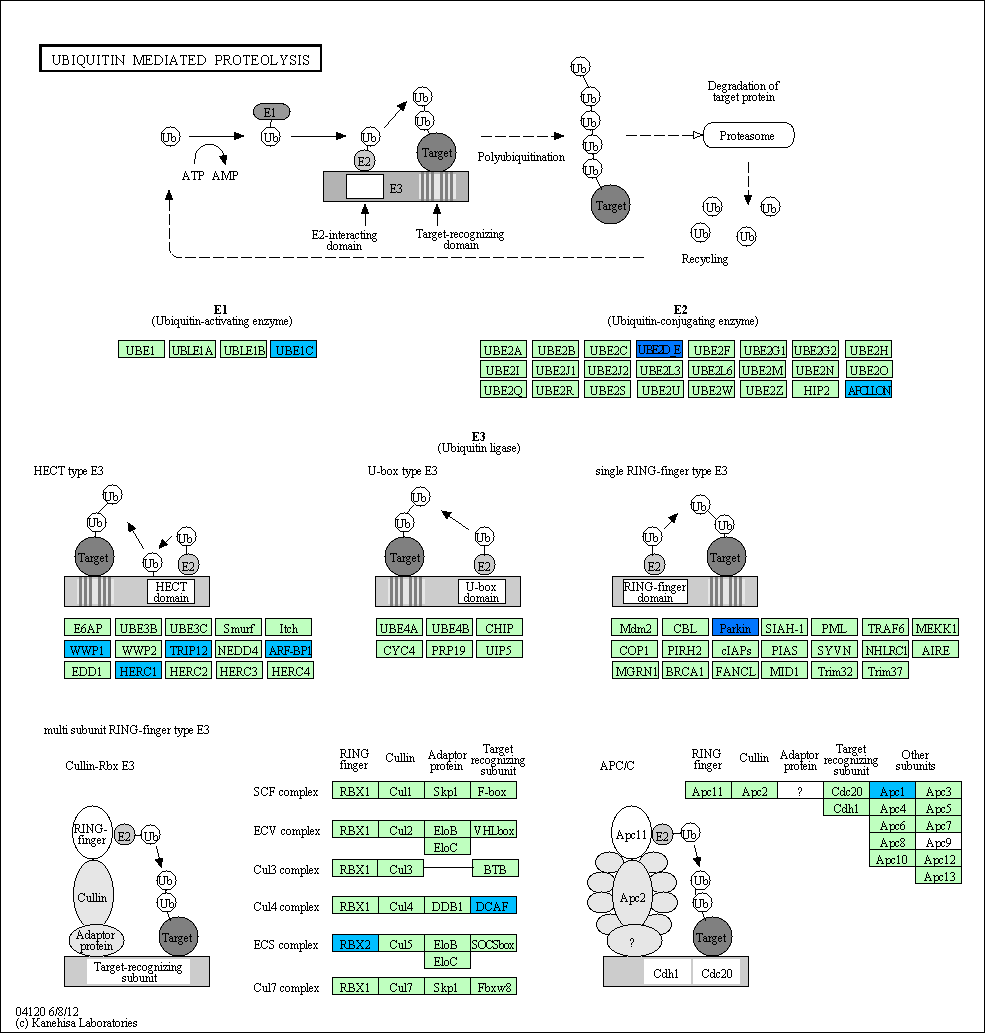

Supplement: Additional file 2: Figure S1 — KEGG Pathway diagram depicting ubiquitin mediated proteolysis. Transcripts enhanced by sleep in the heart are colored light blue. Transcripts enhanced by sleep in both heart and lungs are colored dark blue. [file 1471-2164-14-362-S2.png]

**Lung: 3 Hours**

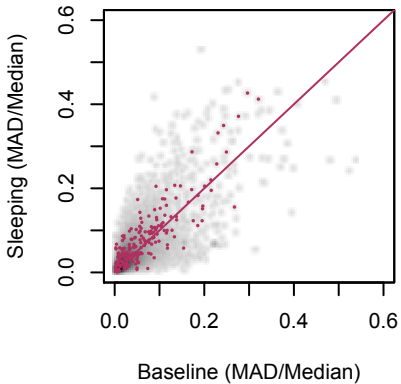

**Lung: 6 Hours**

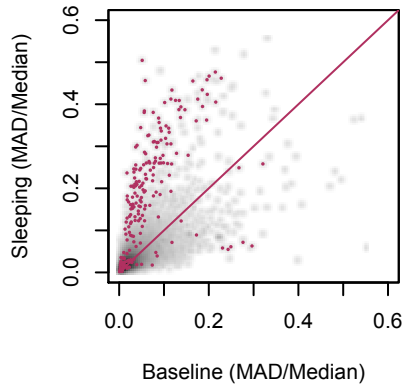

**Lung: 9 Hours**

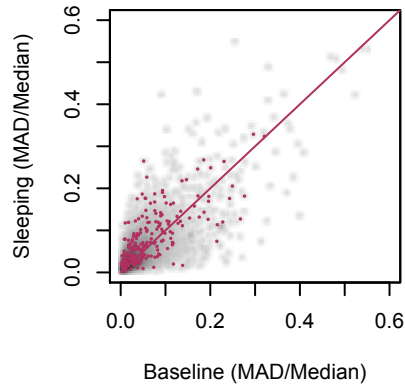

**Lung: 12 Hours**

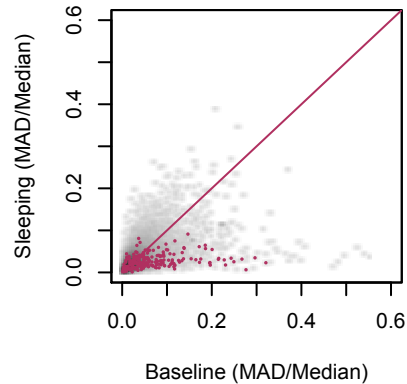

Supplement: Additional file 3: Figure S2 — Effect of sleep and sleep deprivation on the inter-animal variability of transcript expression in the lung as assessed by median deviations. 210 probes showed statistically significant changes in expression variability as a function of sleep duration. The median absolute deviation (MAD) is divided by median expression to obtain a normalized measure of variation resistant to outliers. The normalized MAD among spontaneously sleeping mice after 3, 6, 9 and 12 hours is plotted against the normalized MAD of the same transcript at baseline (lights-on). The red line of identity (x = y) demarcates no change in inter-animal variability. Transcripts with statistically significant temporal changes in variance among sleeping mice were colored in red. As shown in Figure 6A, these transcripts remain clustered and show systematic changes with variability as sleep progresses. [file 1471-2164-14-362-S3.pdf]
